# Chemical Evolution of Natural Product Structure

**DOI:** 10.1021/jacs.1c11270

**Published:** 2022-02-21

**Authors:** Michael Grigalunas, Susanne Brakmann, Herbert Waldmann

**Affiliations:** † Max-Planck-Institute of Molecular Physiology, Otto-Hahn Strasse 11, 44227, Dortmund, Germany; ‡ Faculty of Chemistry and Chemical Biology, TU Dortmund University, Otto-Hahn Strasse 4a, 44227, Dortmund, Germany

## Abstract

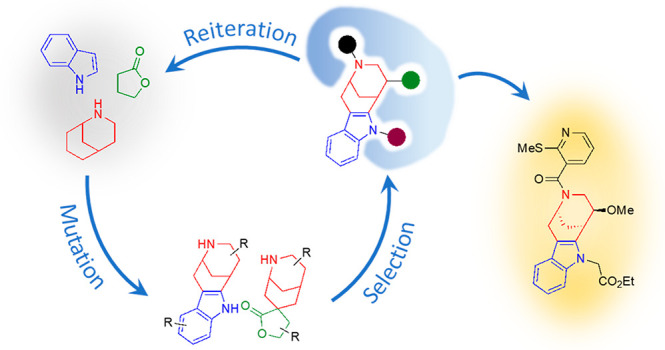

Natural products are the result of Nature’s exploration of biologically relevant chemical space through evolution and an invaluable source of bioactive small molecules for chemical biology and medicinal chemistry. Novel concepts for the discovery of new bioactive compound classes based on natural product structure may enable exploration of wider biologically relevant chemical space. The pseudo-natural product concept merges the relevance of natural product structure with efficient exploration of chemical space by means of fragment-based compound development to inspire the discovery of new bioactive chemical matter through *de novo* combination of natural product fragments in unprecedented arrangements. The novel scaffolds retain the biological relevance of natural products but are not obtainable through known biosynthetic pathways which can lead to new chemotypes that may have unexpected or unprecedented bioactivities. Herein, we cover the workflow of pseudo-natural product design and development, highlight recent examples, and discuss a cheminformatic analysis in which a significant portion of biologically active synthetic compounds were found to be pseudo-natural products. We compare the concept to natural evolution and discuss pseudo-natural products as the human-made equivalent, i.e. the chemical evolution of natural product structure.

## Introduction

The importance of small molecules that can perturb biological systems in a controlled fashion is underpinned by their successful applications in chemical biology^1^ and medicine.^2^ Nevertheless, the continued exploration of biologically relevant chemical space for the discovery of new small molecules remains an essential task for the better understanding of biological processes and for the advancement of therapeutics. This is a challenging endeavor since the possible number of compounds that may qualify as bioactive small molecules is so vast that the time and resources needed to synthesize, let alone biologically evaluate, exceed reality.^3^ Therefore, there is a need for methods and sources of inspiration that can help navigate through chemical space and focus on areas that are biologically relevant.^4^

Through evolution, Nature has been exploring chemical space which is reflected in secondary metabolites or natural products (NPs). Via enzymatic cascades, organisms produce NPs that can carry out specific biological functions either within and/or between organisms to give the producing organism a selective advantage. Based on the demands of the environment, i.e. selectivity pressures, NPs with different molecular scaffolds can be produced that modulate different targets directly or indirectly to provide the organism with a higher level of fitness. Therefore, NPs reflect organisms’ adaptations to their environments through evolution and Nature’s exploration of biologically relevant chemical space. Accordingly, NP structure can be considered biologically prevalidated.

NPs have been and continue to be a reservoir for successful molecular discovery programs;^5^ however, NPs have been subjected to evolutionary constraints. Natural evolution itself is very slow and, in tandem with selection pressures, has resulted in NPs occupying only a fraction of theoretical NP-like chemical space.^6^ These constraints may have led to many known NP scaffolds being highly conserved and therefore the diversity of scaffolds readily accessible from Nature to be limited. Additionally, there are undoubtably numerous biologically intriguing NPs that have not yet been identified. Therefore, solely relying on Nature as a source of biologically active molecules brings about limitations.

Attempts to override the limitations of Nature and expand beyond the chemical space occupied by NPs to provide new small molecules with unexpected or unprecedented bioactivities need to face the question how biologically relevant chemical space can be explored efficiently?^7^

Several chemical design strategies have been developed for the search of biologically active compounds.^8^ Diversity-oriented synthesis (DOS) provides chemically diverse libraries enriched in variations of scaffolds, stereochemistry, and appendages to quickly probe larger amounts of chemical space.^9^ A combinatorial approach utilizing DNA encoded libraries (DEL) has emerged as a method for the generation of relatively large libraries with chemical diversity.^10^ While DOS and DEL can generate expansive libraries that can quickly explore diverse chemical space, more recently in tandem,^11^ and have provided biologically valuable compounds,^12,13^ much of the space explored may not necessarily be biologically relevant.

Several approaches prioritize exploring more focused areas of chemical space that are occupied by NPs of biological interest. Access to this space is facilitated by the design of more synthetically tractable derivatives relative to the parent NP. Compounds resulting from function-oriented synthesis^14,15^ have reduced complexity relative to the parent NP while those from dynamic retrosynthetic analysis proposed by Shenvi et al.^16,17^ retain the intrinsic complexity of the parent NP while minimizing synthetic complexity. While both methods can yield compounds with similar or enhanced biological properties, the chemical and biological space explored is narrow and usually similar to the parent NP’s.

**Figure 1:**
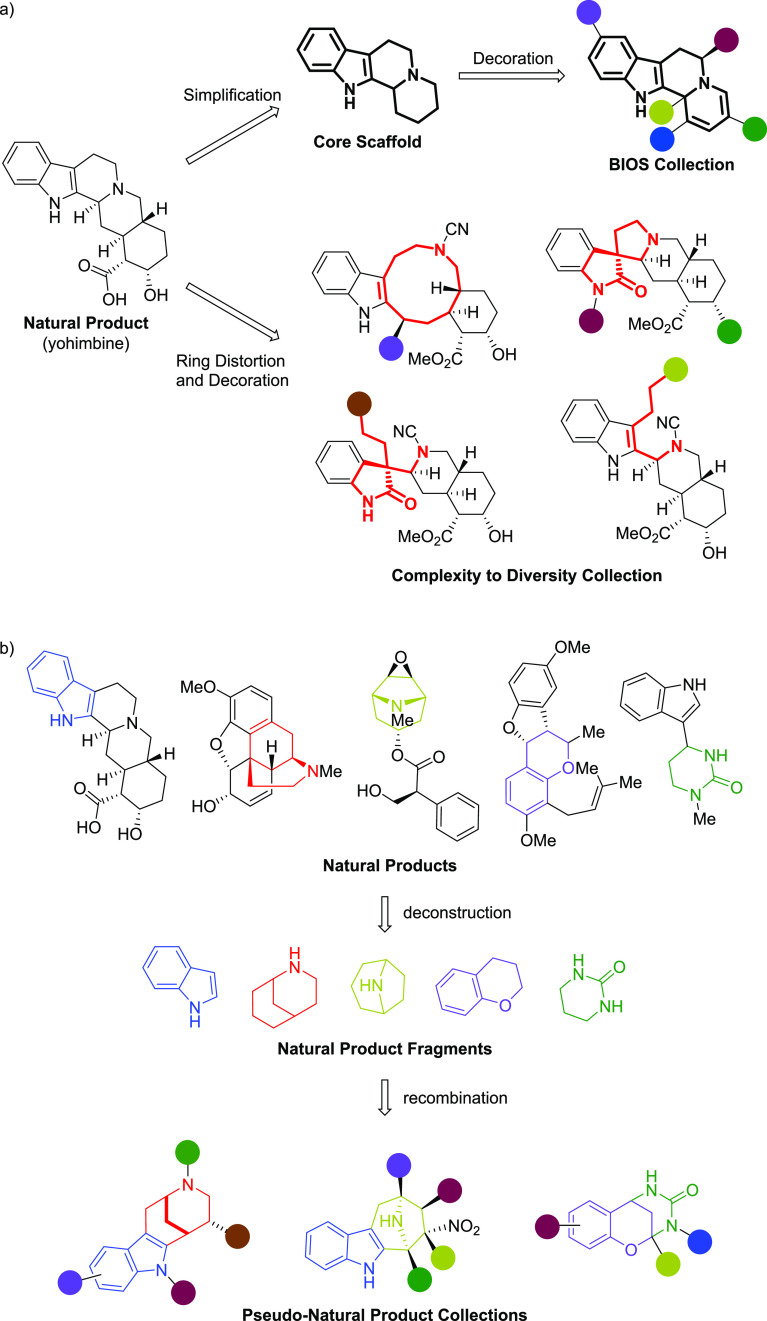
(a) Examples of biology-oriented synthesis^24^ (BIOS) and the complexity to diversity^25^ methods employing the natural product yohimbine as a starting point. (b) Examples of the pseudo-natural product (pseudo-NP) method by computational deconstruction of NPs to NP fragments and *de novo* recombination.^26−28^

Other design principles aim to provide molecules that maintain the biological relevance of NPs by employing logic derived from natural evolution.^18,19^ Biology-oriented synthesis (BIOS) is based on the principle that proteins and NPs have coevolved to encompass only a small portion of chemical space.^20−22^ While appendages of NPs can be highly diverse, the core scaffolds of NPs are conserved as are the corresponding binding pockets of proteins. Using this logic, BIOS employs hierarchical classification to identify simplified NP core structures that may retain biologically relevant characteristics.^23^ Decoration of these scaffolds has led to several biologically active compound collections that retain relevance to NPs but are more synthetically tractable (Figure 1a); however, BIOS is limited both biologically and chemically because the core scaffolds are present in current NPs obtained through existing biosynthetic pathways.

In Nature, common NP intermediates can undergo different biosynthetic pathways to arrive at chemically and biologically diverse NPs as “end points”. The complexity to diversity approach employs “end point” NPs as divergent intermediates and chemically extends biosynthetic pathways by distorting the scaffolds of NPs through ring distortion reactions (Figure 1a).^29−31^ The resulting compound collections have diverse scaffolds that are distinct from the parent NP but retain the complexity of and biological relevance to NPs; however, the concept may be limited by the finite number of possible starting points.

While all of these methods have been successful in providing compound libraries that are enriched in bioactivities, each has, to some extent, limitations that hinder wider exploration of biologically relevant chemical space. To expand beyond these limitations while still retaining relevance to NPs, we have developed the concept of pseudo-natural products (pseudo-NPs).^19,32−34^ The principle merges the biological relevance of NPs with the rapid accessibility to diverse chemical space of fragment-based discovery. Through cheminformatic deconstruction of NPs, we have defined about 2,000 NP fragment groups that represent the chemical space defined by the structures of the NPs known at the time.^35^ The pseudo-NP method aims for the *de novo* combination of NP fragments or NPs that are themselves fragment-sized^35−38^ in a manner that is not observed in current NP structures (Figure 1b). This can be achieved through the mixing of different fragment combinations^39^ and/or the combination of fragments in different orientations.^40^ The new scaffolds retain the chemical and biological relevance of NPs but are not possible through known biosynthetic pathways, hence the name pseudo-NPs.

Notably, the biological goal of pseudo-NP design differs from the synthetic NP-hybridization strategy. The NP-hybridization combines pharmacophores of two NPs that have different but synergistic biological effects. The resulting chimeric molecules can have enhanced therapeutic value but most likely retain the mode of actions and/or biological targets of the two parent NPs.^41−44^ In contrast, the new arrangements of NP fragments in the resulting pseudo-NPs are intended to explore new areas of biologically relevant chemical space and have biological targets that are unrelated to the NP fragments and NPs from which they are derived.

In this perspective, we discuss the design principles and biological evaluation of pseudo-NP collections as well as highlight recent examples. We discuss that, through cheminformatic analysis, pseudo-NPs have been made unintentionally for decades and comprise a significant portion of synthetically made bioactive small molecules. We then tie our studies of pseudo-NPs to the concept of chemical evolution, and argue that the method can be considered the manmade equivalent of NP structure evolution. Taken all together, we argue that the pseudo-NP concept is a valid principle for the wider exploration of biologically relevant chemical space.

## Design and Biological Evaluation of Pseudo-Natural Product Collections

The general design principle of pseudo-NPs revolves around the combination of NP fragments to arrive at scaffolds that resemble NPs but are not possible through known biosynthetic pathways. Pseudo-NPs should share some chemical similarities with NPs in that they are both comprised of NP fragments and should therefore resemble the relatively high fraction of sp^3^ atoms and abundant stereogenicity observed in NP structure.^45,46^ On the other hand, pseudo-NPs should have a degree of chemical distinguishability since fragment combinations and/or orientations in pseudo-NPs are distinct from NPs.

**Figure 2:**
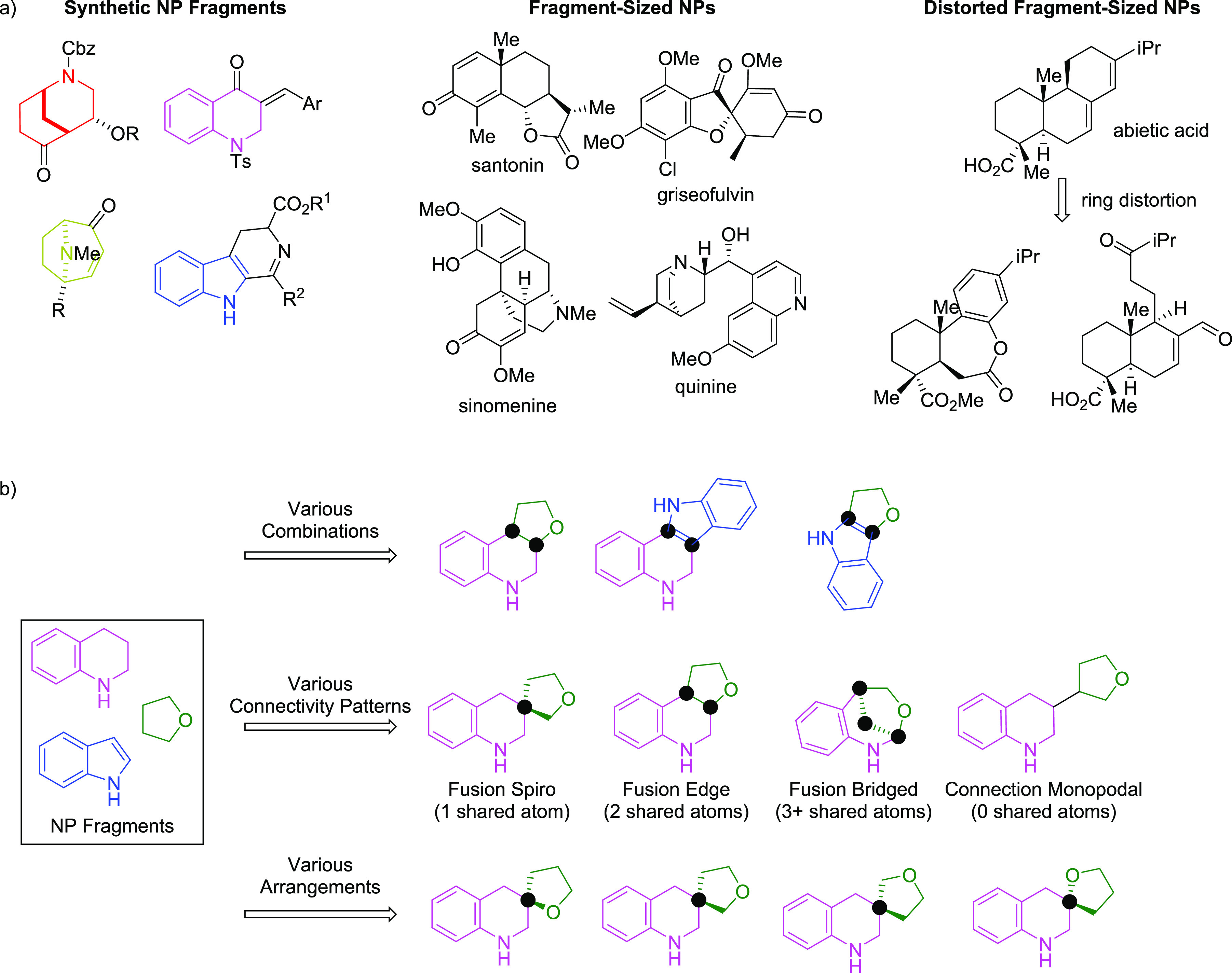
(a) Possible fragment types for combination in pseudo-NP design: synthetic NP fragments, fragment-sized NPs, and ring distorted fragment-sized NPs.^50^ (b) Fragment combination strategies to arrive at diverse pseudo-NPs including mixing fragment combinations, different connectivity patterns, and/or varying regiosomeric arrangements. The black dots indicate common atoms due to the fusion patterns.

The combination partners should be derived from different biosynthetic origins and/or have different heteroatom content (O and N) to ensure the exploration of new chemical space. It should be considered that criteria typically employed by the “rule of three”^47^ for fragments may not be entirely valid for NP fragments.^48^ Therefore, NP-like fragments can be considered to have an AlogP < 3.5, a molecular weight between 120 and 350 Da, ≤ 3 hydrogen bond donors, ≤ 6 hydrogen bond acceptors, and ≤6 rotatable bonds.^35^ Within these criteria, NP fragments can include fully synthetic fragments, NPs that are themselves fragment-sized, and products resulting from the distortion or fragmentation of NPs (Figure 2a).^49^ Suitable NP fragments could have reactive handles that are amenable for the installation of additional NP fragments through complexity generating reactions. Alternatively, pseudo-NP scaffolds and the generation of fragments can be simultaneously constructed through intramolecular reactions.

Chemically diverse pseudo-NP collections can be designed by using different combination strategies (Figure 2b). Novel scaffolds can arise through the mixing and matching of different NP fragments. Additionally, combining similar fragments with different connectivity patterns can add further degrees of variability from known NP structures. Several fragment connectivity patterns are observed in Nature and can be translated to pseudo-NPs. These can be classified into two categories: connections with shared atom(s) and connections without shared atoms. Those with shared atoms include fusion spiro (one shared atom), fusion edge (two shared atoms), and fusion bridged (three or more shared atoms). Fusion patterns without shared atoms include but are not limited to monopodal, bipodal, tripodal, bridged bipodal, and bridged tripodal.^33^ A further degree of scaffold diversification is possible by combining similar fragments with similar connectivity patterns but having different regioisomeric arrangements. Taken together, the combination of different NP fragments with different connection types and/or different regioisomeric arrangements may lead to chemically and biologically diverse pseudo-NP collections.

Since their scaffolds are novel, pseudo-NPs may lead to new chemotypes for known biological targets or to the discovery of unknown biological targets. To efficiently probe broad areas of biological space, biological evaluation of pseudo-NPs should employ unbiased, target-agnostic assays.^51^ We have successfully employed different cell-based phenotypic assays to identify pseudo-NPs that affect important cellular processes or signaling cascades, including monitoring glucose uptake, autophagy, Wnt and Hedgehog signaling, T-cell differentiation, and induction of reactive oxygen species.

Beyond phenotypic assays, morphological profiling via the “Cell Painting Assay” can be used to evaluate compound-induced morphological changes in the entire cell.^52,53^ In the Cell Painting Assay, images of treated cells are acquired through fluorescent microscopy followed by the extraction of several hundred features to provide a characteristic “fingerprint” that encompasses the cells’ morphological changes. Compound-induced morphological perturbation can be quantified as an “induction value” by calculating the number of significantly changed features relative to a DMSO control. Compounds that have suitable induction values are considered bioactive. The fingerprints can also be compared to reference compounds with known bioactivities to generate target/modes of action hypotheses or compared within and between compound collections for biological performance.

## Hybrid Natural Product Strategies are Employed by Nature

**Figure 3:**
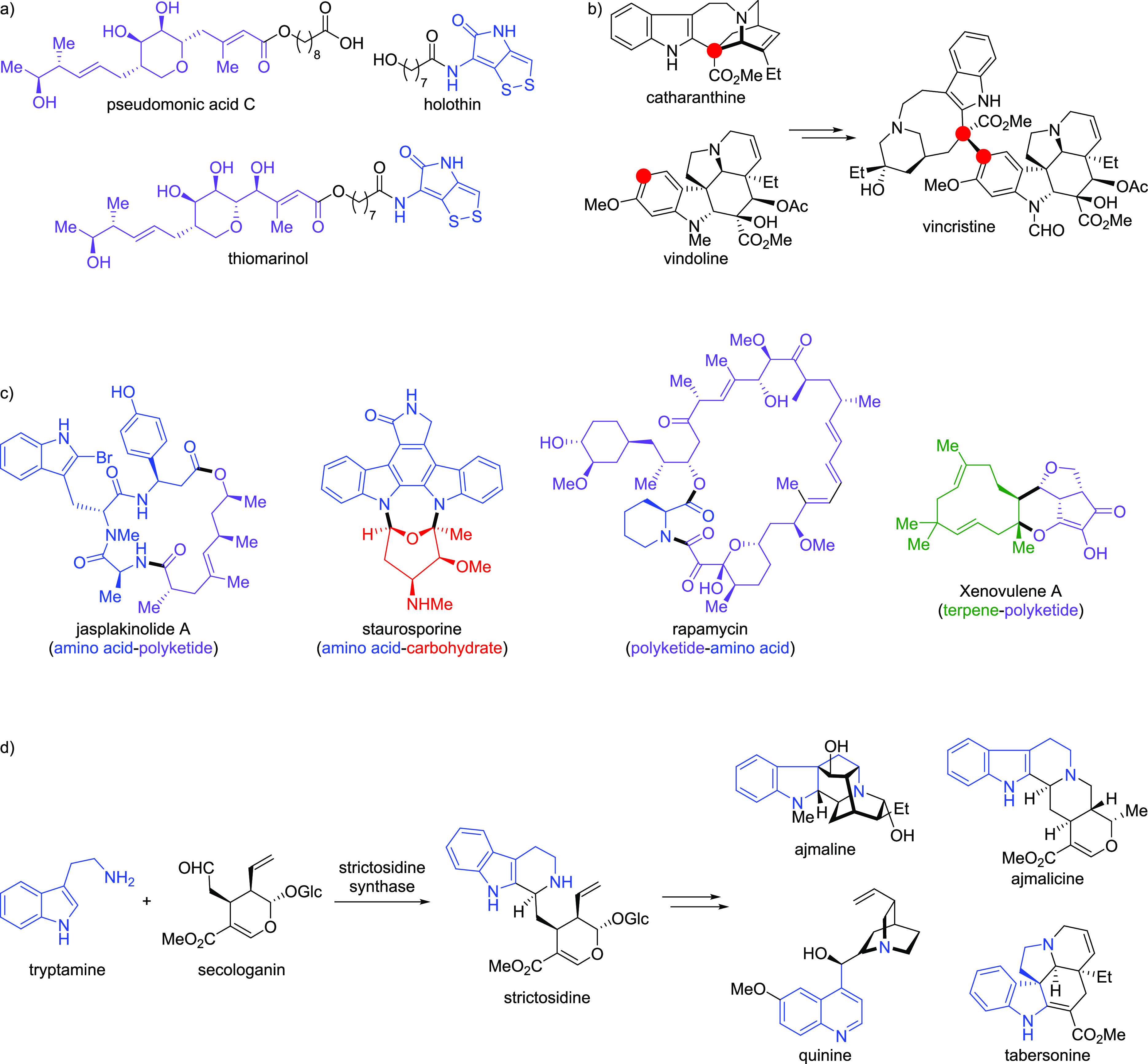
Naturally occurring hybrid natural products. (a) The hybrid natural product thiomarinol resembles the combination of pseudomonic acid C and holothin. (b) The biosynthesis of vincristine includes the heterodimerization of the natural products catharanthine and vindoline. Points of connectivity are highlighted in red. (c) Selected hybrid natural products that are the combination of more than one metabolic unit of different biosynthetic origin. (d) Representative monoterpene alkaloids from the divergent biosynthetic intermediate strictosidine. Strictosidine is the combination of the amino acid derivative tryptamine and the monoterpene glycoside secologanin. Atoms from tryptamine are highlighted in blue.

The concept of combining privileged NP scaffolds, i.e. fragment-sized NPs and/or NP fragments, to provide “hybrid NPs” has also been explored by Nature. Hybrid NPs may be characterized as the product of homo- or heterodimerizations of smaller NPs in which the monomeric NPs could be, but are not necessarily, from different biosynthetic origins.^43^ Dimerized NPs are typically combined via a monopodal connection and retain resemblance to the core scaffolds of the monomeric NPs, such as thiomarinol and vincristine. The structure of thiomarinol resembles the combination of biosynthetic derivatives of the antibiotics pseudomonic acid C and holothin (Figure 3a).^54,55^ While the monomers represent two separate classes of antibiotics, their incorporation into what is the scaffold of thiomarinol gives a more potent, broader spectrum antibiotic. This enhanced antibiotic acitivity may be explained by the antibiotic properties of the two monomers being retained in thiomarinol, resulting in a synergistic effect. The antimitotic drug vincristine is used for the treatment of leukemia and lymphomas^56^ and is the combination of the indole alkaloids catharanthine and vindoline (Figure 3b).^57^ Interestingly, catharanthine and/or vindoline induce mitotic arrest only at concentrations several orders of magnitude greater than vincristine.^58^ The significant difference in potency of vincristine may be rationalized by a binding mode to tubulin that is not possible with solely the monomers.^59^

Other types of hybrid NPs fuse biosynthetically unrelated metabolic units (or fragments),^60^ including but not limited to amino acids, carbohydrates, polyketides, and terpenoids, to form the hybrid NP core scaffold (Figure 3c).^61−64^ These compounds offer diversity through different fragment combinations that biosynthetic pathways of a single origin cannot provide. After combination, biosynthetic cascades can further distort and fuse the original metabolic units into complex scaffolds that do not intuitively resemble the original pieces. Strictosidine is formed through the combination of the amino acid derivative tryptamine and the monoterpene glycoside secologanin and is the biosynthetic divergent intermediate for thousands of monoterpene alkaloids with complex multipodal fusion patterns (Figure 3d).^65^

Where these two approaches diverge is in the preparation of new molecules. Nature synthesizes NP hybrids through enzymatic reactions that are summarized as biosynthetic pathways. Through evolution, new biosynthetic pathways can arise that may yield new NP hybrids. On the other hand, pseudo-NPs are designed by deconstruction of NPs to NP fragments through cheminformatic analyses. De novo recombinations are envisioned and synthesized via chemical reactions that may not occur in Nature.

Both methods may produce biologically relevant chemical matter; however, evolution of biosynthetic pathways in Nature to provide new molecules is slow while synthesis allows this process to be accelerated and more flexible. By employing chemical synthesis, reaction pathways can be employed that are not possible through biosynthesis resulting in new combinations and connectivities of NP fragments. Therefore, the pseudo-NP concept does not necessarily compete with NP hybridization but rather complements it and explores areas of biologically relevant chemical space that are not possible through existing biosynthetic pathways.

## Recent Examples of Pseudo-Natural Products

### Indofulvins

**Figure 4:**
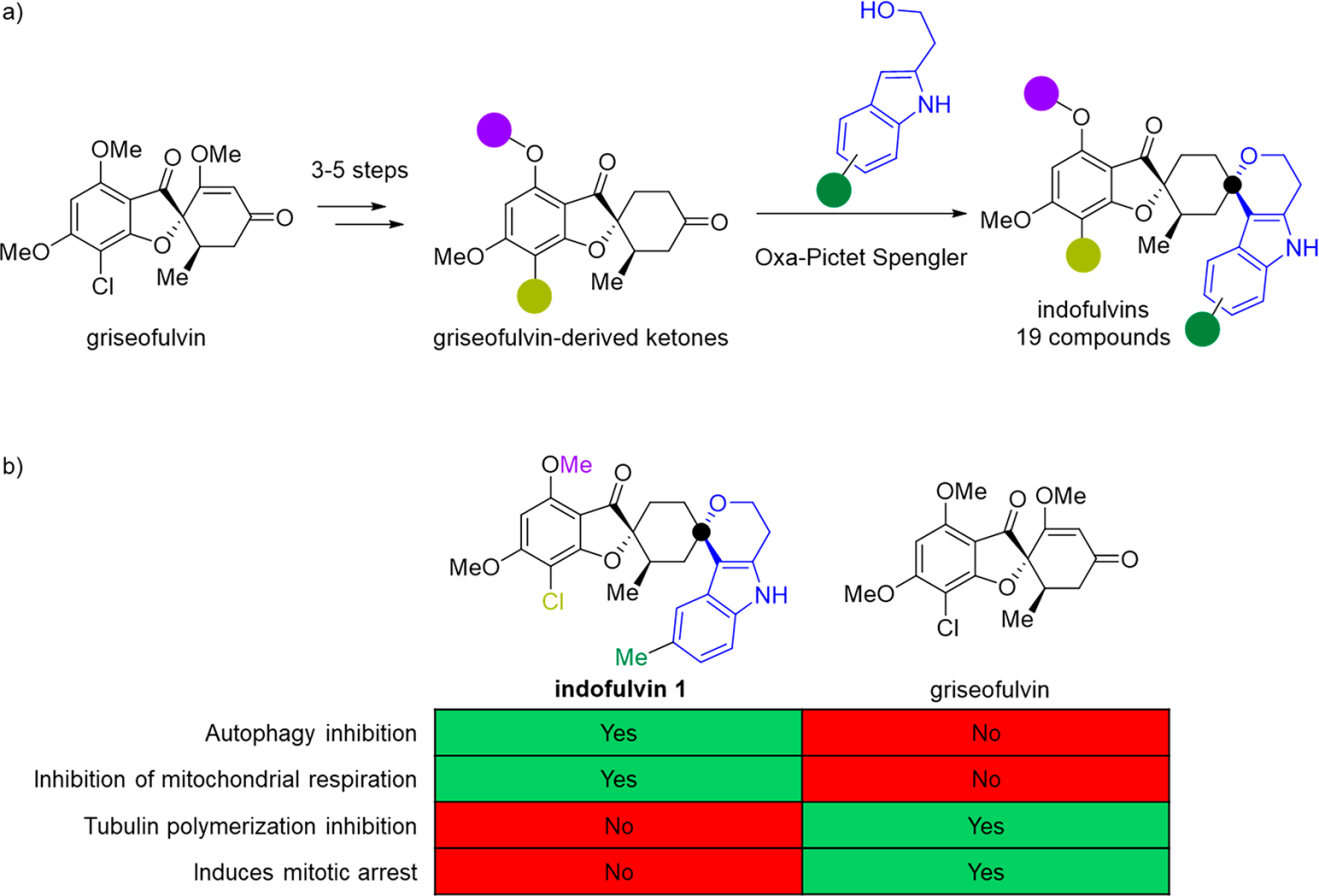
(a) Synthesis of the indofulvin pseudo-NP collection through the combination of the fragment-sized NP griseofulvin and 4*H*-pyranoindole derivatives. (b) Biological comparisons of the pseudo-NP **indofulvin 1** and griseofulvin showing that the combination of fragments has led to new bioactivity of autophagy inhibition potentially through mitochondrial respiration while simultaneously losing the native tubulin-affecting bioactivity of griseofulvin. The black dot indicates a common atom due to the fusion pattern.

A recent example of the pseudo-NP concept combined the indole-containing fragment 4*H*-pyranoindole and the fragment-sized NP griseofulin to arrive at indofulvins (Figure 4a).^66^ A collection of 19 indofulvins was obtained by employing an iso-oxa-Pictet Spengler (IOPS) reaction as the key step to combine the two fragments with a fusion spiro connection pattern. While the Pictet Spengler reaction is present in biosynthetic pathways, its iso-version is not currently in the biosynthetic repertoire, resulting in a novel arrangement of the 4*H*-pyranoindole fragment.

Biological evaluation of the collection in various phenotypic assays revealed that indofulvins are inhibitors of starvation-induced autophagy, with **indofulvin 1** being the most potent compound (IC_50_ = 0.82 μM). While **indofulvin 1** inhibited autophagy and was validated at a protein level, neither griseofulvin nor the 4*H*-pyranoindole fragment of **indofulvin 1** affected autophagy (Figure 4b). Evaluation of **indofulvin 1** via the Cell Painting Assay revealed a high fingerprint similarity to oligomycin which targets mitochondrial respiration and led to the hypothesis that **indolfulvin 1** may inhibit autophagy through a similar mode of action. In a Mito Stress Test,^67^**indofulvin 1** dose-dependently reduced the oxygen consumption rate and increased the rate of extracellular acidification and suggests that **indofulvin 1** may inhibit autophagy via modulation of mitochondrial function.

Griseofulvin is known to modulate tubulin and cause mitotic cellular arrest. To see if the inherent bioactivity is retained, griseofulvin was compared to **indofulvin 1** in a tubulin polymerization assay and in a cell-based assay measuring mitotic arrest. Griseofulvin showed dose-dependent activity in both assays whereas **indofulvin 1** was inactive and led to the conclusion that **indofulvin 1** does not retain the native bioactivity of griseofulvin.

Overall, the indofulvin class represents an unprecedented chemotype for autophagy inhibition. This example indicates that the combination of fragments can provide new bioactivity while simultaneously losing the native bioactivity of an initial fragment and adds validation to the underlying principle of pseudo-NPs.

### Sesquiterpenoid Alkaloids

**Figure 5:**
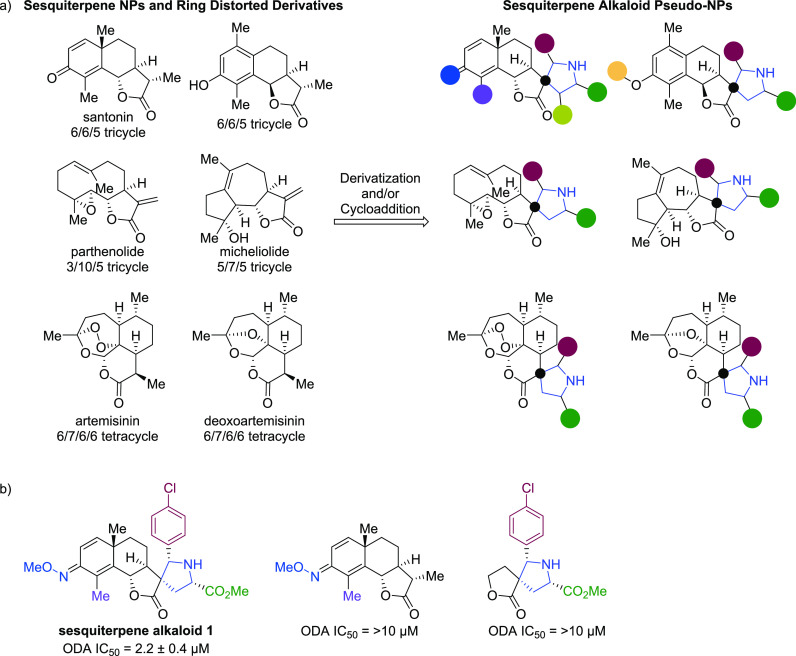
(a) Combination of sesquiterpene NPs and ring distorted derivatives with a pyrrolidine fragment to arrive at a chemically and stereochemically diverse collection of sesquiterpene alkaloid pseudo-NPs. (b) Identification of **sesquiterpene alkaloid 1** as an inhibitor in a Hedgehog-dependent osteoblast differentiation assay (ODA).^69^ The individual Santonin-derived and pyrrolidine fragments were inactive in an ODA at 10 μM. The black dots indicate common atoms due to the fusion patterns. The colored circles in Figure 5a represent points of derivatization and correlate to the substitutions in Figure 5b.

A diverse collection of compounds was produced by merging the principles of complexity to diversity and pseudo-NPs.^68^ The strategy employed six fragment-sized sesquiterpenoid lactones that are either NPs or were obtained via ring distortion and can be considered to be biosynthetically related scaffolds but are yet chemically diverse (Figure 5a). The pseudo-NP principle was then applied by combining the sesquiterpenoid fragments with the biosynthetically unrelated alkaloid fragment pyrrolidine via a 1,3-dipolar cycloaddition to arrive at sesquiterpenoid alkaloid pseudo-NPs. The versatility of the cycloaddition allowed for access to various pyrrolidine diastereomers based on the ligand and conditions employed. In total, 89 chemically and stereogenically diverse sesquiterpenoid alkaloid pseudo-NPs were synthesized.

The morphological diversity of the collection was compared via the Cell Painting Assay. The Cell Painting Assay fingerprints of the sesquiterpenoid alkaloid pseudo-NPs and sesquiterpenoid lactone derivatives were clearly distinguishable, indicating that the addition of the pyrrolidine fragment had changed the bioactivity. Different pyrrolidine stereoisomers of dehydrosantonin-derived pseudo-NPs could be identified by the Cell Painting Assay, representing biological diversity through different diastereomers; however, when keeping the pyrrolidine fragment constant and comparing different sesquiterpenoid fragments, the results were not as clearly defined. While some compounds had a low chemical similarity, the similarity of their fingerprints in the Cell Painting Assay was relatively high. Nevertheless, the common pyrrolidine fragment was found not to be solely responsible for the high fingerprint similarities, as this fragment by itself did not share the similar fingerprint to the pseudo-NPs.

The library was also subjected to various phenotypic assays, and **sesquiterpene alkaloid 1** was identified as a new chemotype for the inhibition of Hedgehog-dependent osteoblast differentiation (Figure 5b). Interestingly, three other diastereomers as well as the two partial structures of the active compound were inactive at 10 μM indicating that it is the combination of fragments that gives rise to the pseudo-NP’s bioactivity. Together, these results suggest that merging the complexity to diversity and pseudo-NPs concepts may be complementary and lead to chemically and biologically diverse compound classes that are not accessible through existing biosynthetic pathways. We have also successfully synthesized and biologically characterized several other pseudo-NPs^33^ which are depicted below in Figure 11.

## Many Synthetic Bioactive Compounds are Pseudo-Natural Products

A pseudo-NP can be generally defined as a synthetic or biosynthetically engineered small molecule that contains two or more NP fragments in arrangements that are not found in Nature. Although the design principles for the pseudo-NP concept have only recently been outlined, many bioactive compound classes that qualify as pseudo-NPs may have been unintentionally synthesized over the years. In order to shed light on the frequency of pseudo-NPs, NP fragment combinations and connectivity patterns within the Dictionary of NPs (DNP), representing NPs, and synthetic, non-natural compounds in the ChEMBL database, representing synthetic bioactive compounds, were cheminformatically analyzed and compared.^70^ NP fragments were defined from a previous study^35^ and further refined to 1,674 Murcko scaffolds. Due to its abundance in both NPs and synthetic compounds, the benzene fragment was excluded from these studies. NP fragment connectivities were also evaluated and resulted in the consideration of 18 plausible connectivity patterns; however, only the five most frequent connectivity patterns will be discussed here.

Analysis of curated DNP and ChEMBL databases revealed that both have a similarly high percentage of compounds that contain two or more NP fragments (DNP = 67%, ChEMBL = 61%). Furthermore, 344,394 (21%) compounds in the curated ChEMBL data set fit the definition of pseudo-NPs. These pseudo-NPs are predominantly comprised of 2–4 NP fragments that are combined by 5 different connectivity patterns. Overall, the ChEMBL pseudo-NPs are highly diverse and have 117,000 unique scaffolds with <100 members per scaffold.

Since there is a high proportion of pseudo-NPs in the ChEMBL database, a literature search was conducted to see their historical trends. It was found that pseudo-NPs have been made continuously for over 40 years, and that the percentage of pseudo-NPs in published structures per year has been increasing over time. These studies indicate that a significant portion of bioactive synthetic compounds are pseudo-NPs and have been synthesized without the pseudo-NP concept as a guiding principle, further adding validation to the overall concept.

### Comparison of Natural Product Fragments, Connectivity Patterns, and Molecular Properties in Synthetic Pseudo-Natural Products and Natural Products

**Figure 6:**
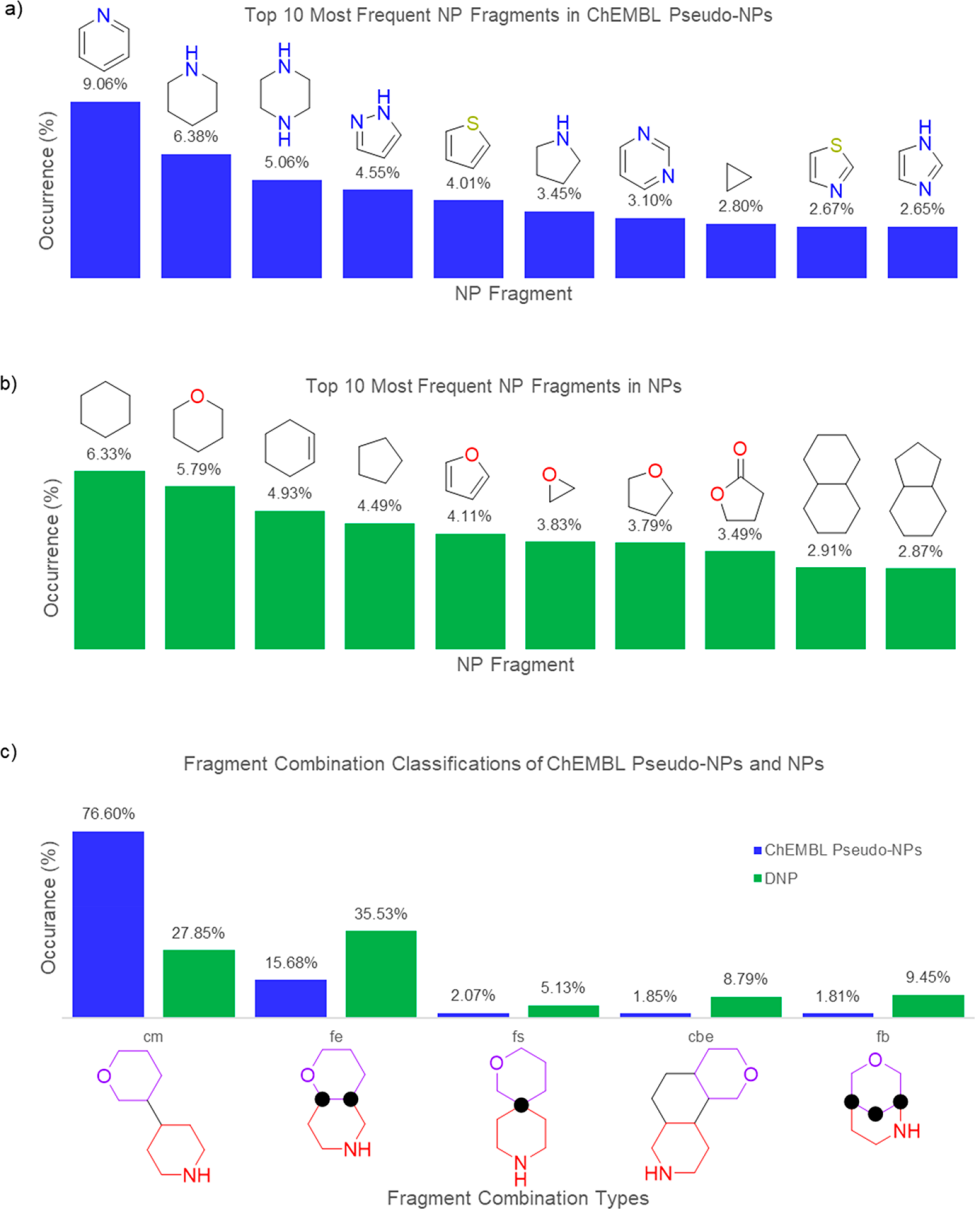
Top ten most frequent NP fragments in (a) ChEMBL pseudo-NPs and (b) NPs.^70^ (c) Occurrence of fragment combinations of ChEMBL pseudo-NPs (blue) and NPs (green).^70^ Examples of connectivity types are depicted below the graph with the NP fragments in purple and red. The black dots indicate common atoms due to the fusion patterns. cm = connection monopodal; fe = fusion edge; fs = fusion spiro; cbe = connection bipodal edge; fb = fusion bridged.

While pseudo-NPs frequently populate the ChEMBL database, the ChEMBL pseudo-NPs differ significantly from NPs for which NP fragments are combined and how they are combined. The most abundant (top 10) NP fragments observed in the ChEMBL pseudo-NPs are nitrogen- and sulfur-containing heterocycles, with more than half of them being aromatic (Figure 6a).^70^ On the other hand, the DNP set is enriched in alicyclic and oxygen-containing ring systems with only one aromatic fragment in the top 10 (Figure 6b). Trends in NP fragment connection types are also different between the two sets. ChEMBL pseudo-NPs are dominated by the monopodal connectivity (cm, 77%) whereas NPs’ connectivity types are more broadly distributed and are dominated by multibond connectivities (Figure 6c).

The molecular features also varied significantly between data sets (Table 1 and SI Figure 1). NPs are more enriched in oxygen content while ChEMBL pseudo-NPs have a higher abundance of nitrogen atoms. Furthermore, the fraction of sp^3^ hybridized atoms in NPs is significantly higher than ChEMBL pseudo-NPs. NP-likeness scores^71^ of the two data sets were calculated in which a higher value indicates the set is more NP-like. Even though the ChEMBL pseudo-NPs are comprised of NP fragments, the NP-likeness score was significantly lower than that for the DNP data set. The differences in molecular features and NP-likeness scores may reflect the frequency with which fragments are used and how they are combined. The realization of these trends may aid in the rational design of future bioactivity-enriched pseudo-NP collections and is expanded upon in the Discussion section.

**Table 1:** Molecular Features and NP-Likeness Scores of ChEMBL Pseudo-NPs and DNP Reference Sets^a^

	ChEMBL Pseudo-NPs	DNP
Molecular Weight	412.77 ± 103.09	419.98 ± 146.39
Number of O Atoms	2.48 ± 1.77	5.75 ± 3.78
Number of N Atoms	4.11 ± 1.83	0.58 ± 1.27
fsp^3^	0.33 ± 0.18	0.63 ± 0.24
NP-Likeness Score	–1.10 ± 0.77	2.10 ± 0.86

a Values are averages of the data sets. An NP-likeness score^71^ was used to compare atom connectivities. A higher NP score is more NP-like while a lower score is less NP-like. fsp^3^ = fraction of sp^3^ hybridized atoms. DNP = Dictionary of Natural Products. Plots for molecular feature distributions in both curated data sets can be found in the Supporting Information (SI Figure 1).

## Pseudo-Natural Products as a Strategy for the Chemical Evolution of Natural Product Structure

Evolutionary lines of thought have become part of the chemical mindset at the latest since the introduction of combinatorial synthetic principles for the discovery of new biologically active compounds and therapeutics. The power of *natural evolution*, however, reaches far beyond combinatorial reaction design and the extensive collection of molecules in libraries. Central to natural evolution is the fact that *information* of properties that determine an organism’s fitness in a given environment is passed on to descendants and, by mutation and selection, continually extended and improved. We discuss here that this important idea can be applied to the design and improvement of pseudo-NP structures. To this end, a precise definition of the term “information” in a chemical context is required.

Natural evolution relies on *genetic information* which, on the molecular level, is contained in DNA or RNA and encoded by molecular symbols, i.e. nucleobases A, G, C, T (U). The totality of genetic information or hereditary makeup of an organism is called *genotype*. It is translated into a totality of characteristics, called *phenotype*, that results after expression of genetic information in the form of proteins. These enable a wide repertoire of functions because of their ability to fold into three-dimensional structures and having a greater spectrum of specific chemical properties than nucleic acids.

**Figure 7:**
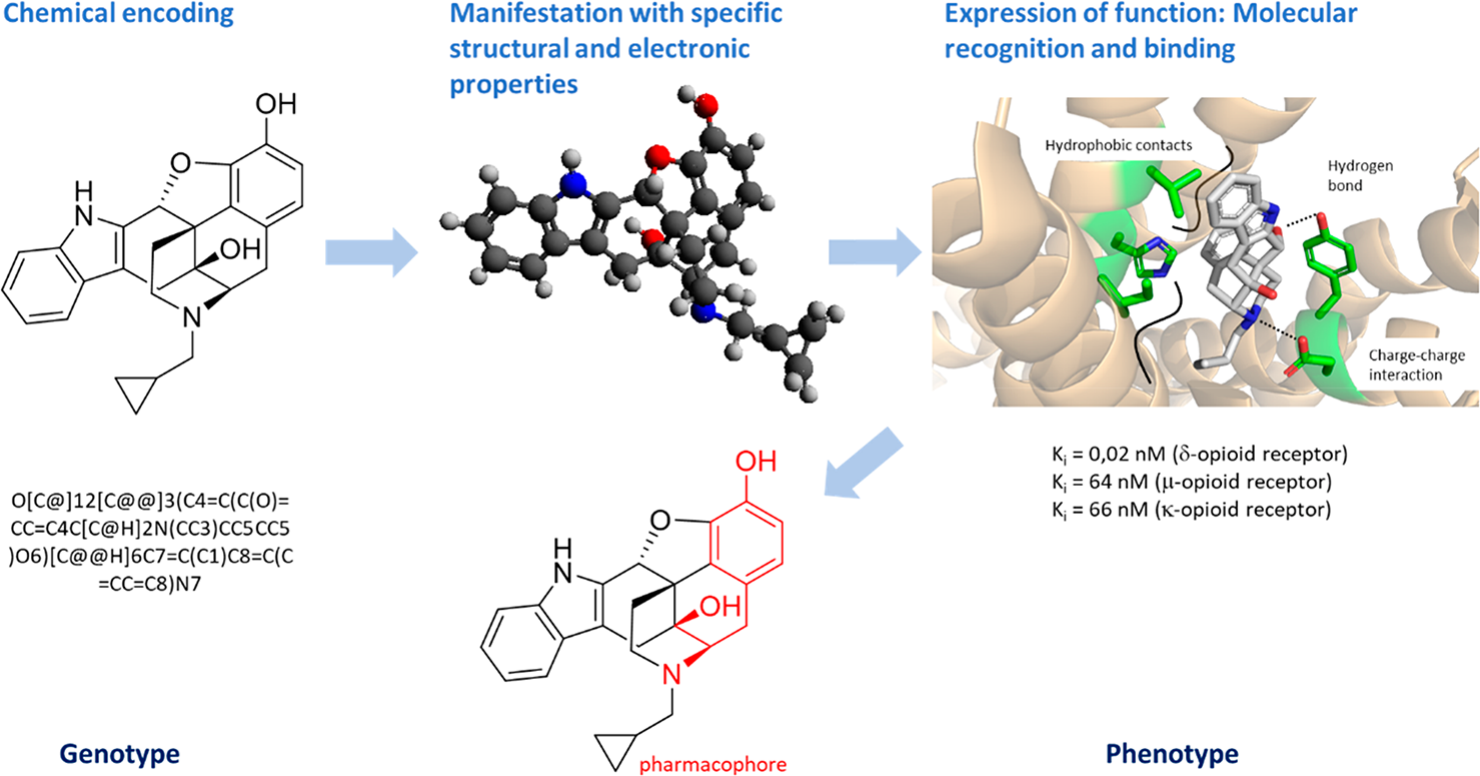
Left, representation of naltrindole, an opioid receptor antagonist using standard encoding by molecular graphics or a SMILES code. Both codes represent the chemical information on the molecule and at the same time its genetic information, or its genotype, which may be inherited and mutated during chemical synthesis. Center, the three-dimensional representation of naltrindole puts functional groups in perspective; these may interact with complementary structures, e.g. in a receptor protein, and can (*a posteriori*) be associated with pharmacophore parts of the molecule. Right, naltrindole can recognize and bind to diverse opioid receptors via electrostatic interactions, hydrogen bonds, and hydrophobic interactions thereby exerting its function (here, inhibition). Protein structure: **4ej4**.^76^

Molecules in general contain *chemical information*, and molecular structures are depicted (or *encoded*) by two-dimensional representations and sometimes by additional codes such as SMILES. Additionally, a molecule can be described by a number of variables which include the microenvironment of every single atom such as the number of its valence electrons, bonds, non-H atoms, and specific symmetry properties. The chemical information on this molecule then results from the sum of these variables (for a detailed description, see Böttcher^72^ and Demoret et al.^73^). The term “information”, however, is of value only with the assignment of an information content or a *meaning*. In Nature, the meaning of a gene becomes evident after translation into a protein with the ability to perform a defined task, for example, as an enzyme. A protein will affect the quality of life, or *fitness*, of an organism which is continually evaluated by natural evolution. For small molecules, two-dimensional projections correspond to structures with spatial and electronic properties; these express a meaning based on the fact that they can interact (as a ligand, agonist, or antagonist) and react with other molecules. These *functions* may be observed e.g. with pharmacological or biochemical properties such as inhibitory activity (K_i_) or substrate quality (K_M_) and, similar to the function of a protein, be targeted by evolution—in Nature or experiment.^74^ Evolution of small molecule structure (more specifically, natural product structure) and function during chemical synthesis, however, requires that the chemical information describing its properties and its potential to interact can be *inherited* to derivatives and undergo *mutation*, i.e. by modification of its molecular symbols (atoms, fragments, etc.). In this sense, chemical information is genetic information. In congruence with definitions for “genotype” and “phenotype” that were originally derived for a molecular description of evolution by M. Eigen,^75^ the structure of a molecule can be viewed as its *genotype*, and the function it performs as its *phenotype* (for an example, see Figure 7).

From the medicinal chemist’s point of view, the information contained in NP structures can also be described as *biologically relevant* because NPs exert effects on the reproduction rate of an organism that is evaluated during evolution. As explained above, genetic information can be inherited, mutated, and selected. In Nature, the term “mutation” refers to a range of mechanisms from substitution of a single monomeric symbol to recombination, duplication, or deletion of genes. Experimentally, these processes can be realized using individual or combinatorial synthetic and biosynthetic strategies.

**Figure 8:**
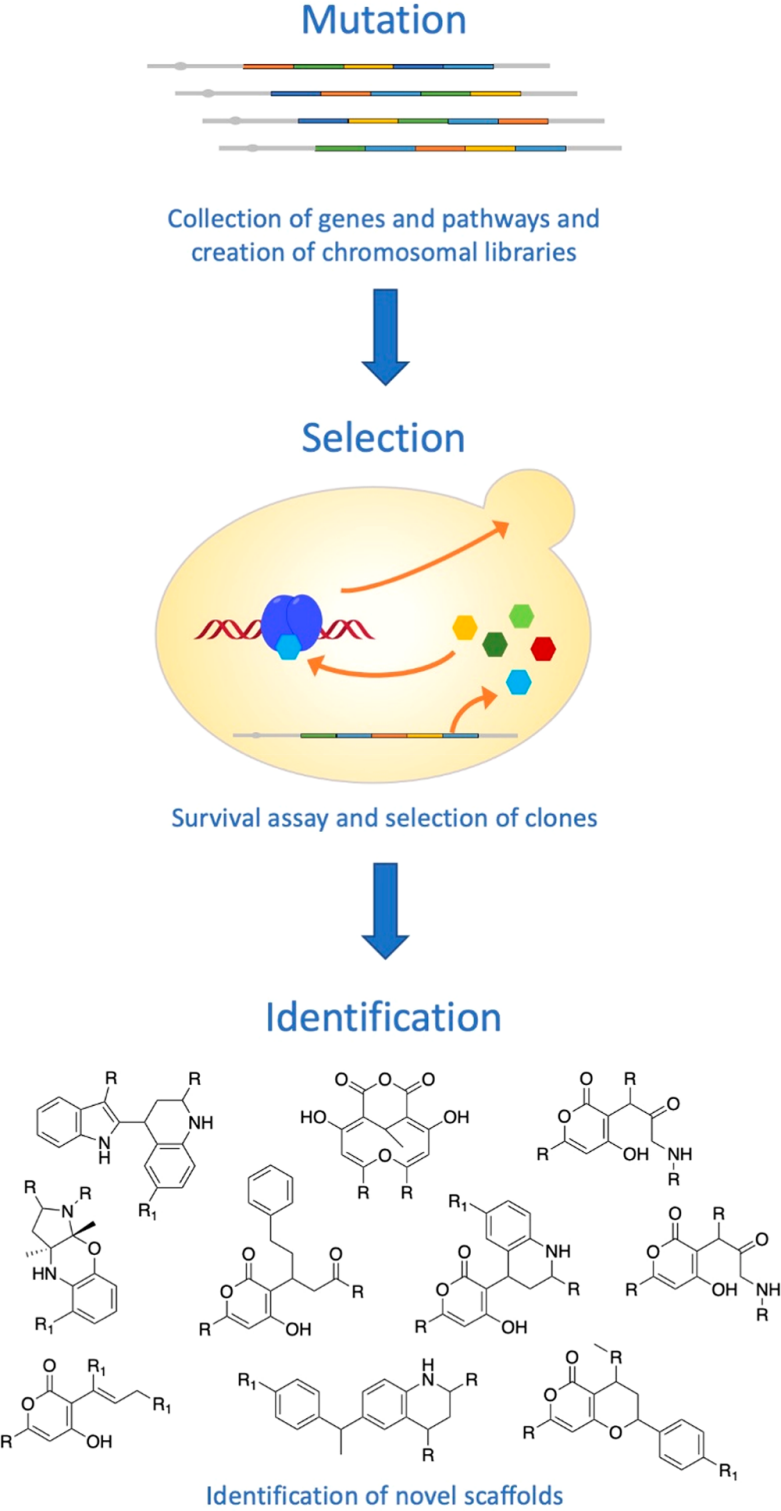
Scheme of the combinatorial approach developed by Evolva.^77^ Genetic information encoding biosynthetic pathways or single enzymes was collected from diverse natural sources and recombined using molecular cloning techniques. Expression of these genes and gene combinations in a microbial host allowed for the alteration or supplementation of existing pathways, thereby enabling the synthesis of new or modified natural products. Their presence was challenged in a cellular assay in which surviving clones showed altered or even “fitter” behavior. These clones were isolated, sorted according to selective criteria, and submitted to detailed analyses for identifying small molecules.

An example for accessing previously unknown NPs that combine known fragments in ways not observed before was recently demonstrated in a landmark study that employed genetic material encoding enzymes and enzymatic cascades from a collection of natural sources (Figure 8).^77^ Genes were recombined using DNA cloning techniques and expressed in a microbial host which then was cultivated in a “survival assay” (i.e., requiring the action of certain target proteins). As a result, 74 novel structures were identified of which more than 75% had not been described previously. Analysis revealed that some combinations of substructures emerged for the first time while other molecules showed known substructure combinations but with *new connectivities*. It can be concluded that the recombination of biosynthetic pathways, as shown in this study, leads to new linkages of the symbols (here, NP fragments) and thereby to the encoding of new information by new structures. A similar statement has been made by Shenvi for new encoding by chemical synthesis.^73^

Nature’s potential to mutate, recombine, and select that leads to hybrid NP structures as described above can be combined with insights into the informational background and biosynthetic mimicry of evolutionary processes. To meet this goal, NP fragments can be considered as building blocks with inheritable genetic information which can not only be combined according to “Nature’s state-of-the-art” but also by use of a repertoire of synthetic strategies. Thereby, new and unprecedented connectivities may be explored, which may not be possible solely based on natural (biosynthetic) reactions. This requires reaction mechanisms that reflect all possible electronic and geometric degrees of freedom of the respective fragments.

**Figure 9:**
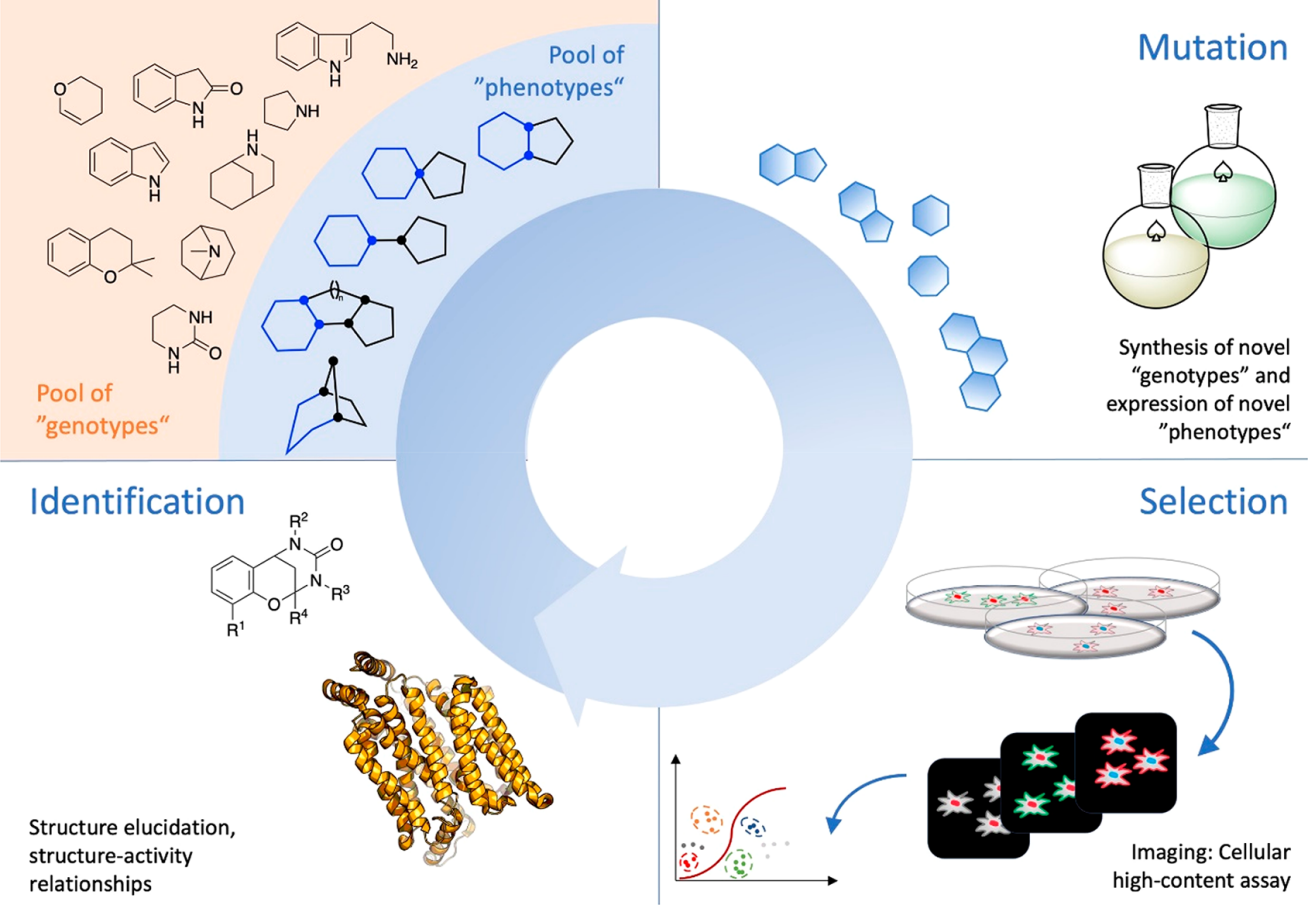
Focused chemical evolution. A “pool of genotypes” is synthesized starting from a set of NP fragments using synthetic strategies that allow for the formation of specific connectivities and thus, the expression of “phenotypes” which may be further derivatized. Due to structural properties such as the number of sp^3^-hybridized atoms, stereogenic atoms, heteroatoms, and aromaticity, the phenotypes exhibit varying potentials to recognize and interact with proteins, e.g. enzymes. This will become evident upon application to cellular screening platforms which can be monitored for structural changes, e.g. by fluorescence imaging. A variety of data can be observed, combined, and sorted according to desired criteria. Small molecules, which cause a relevant change, are isolated, characterized, and submitted to studies of structure–activity relationships, thereby revealing new chemical information. The outcome is a starting point for successive rounds of mutagenizing synthesis, selection, and identification. For definitions of genotype, phenotype, evolution, and information in different contexts, please see the Supporting Information.

The pseudo-NP strategy requires that NP fragments (= inheritable genotypes) are varied by mutation (substitution, derivatization) and recombination (fragment assembly). In a combinatorial synthetic setting, the pool of fragments will be combined and result in a pool of PNPs with new three-dimensional structures that open up new possibilities to recognize, bind, and react (= new phenotypes). These can be tested for novel or desired bioactivities using appropriate target molecules or cellular systems then identified and categorized with regard to structure–activity-relationships, mode-of-action, and putative targets. The outcome of such a cycle of variation (mutation) and identification (selection) is, besides the new molecule(s), new chemical and biological information that can be used as input for another cycle (Figure 9). In conclusion, the complete iterative process of (1) designing, (2) preparing, and (3) biologically characterizing mimics the Darwinian view of evolution from “mutation–selection–amplification” and can be regarded as a chemical evolution of NP structure.

**Figure 10:**
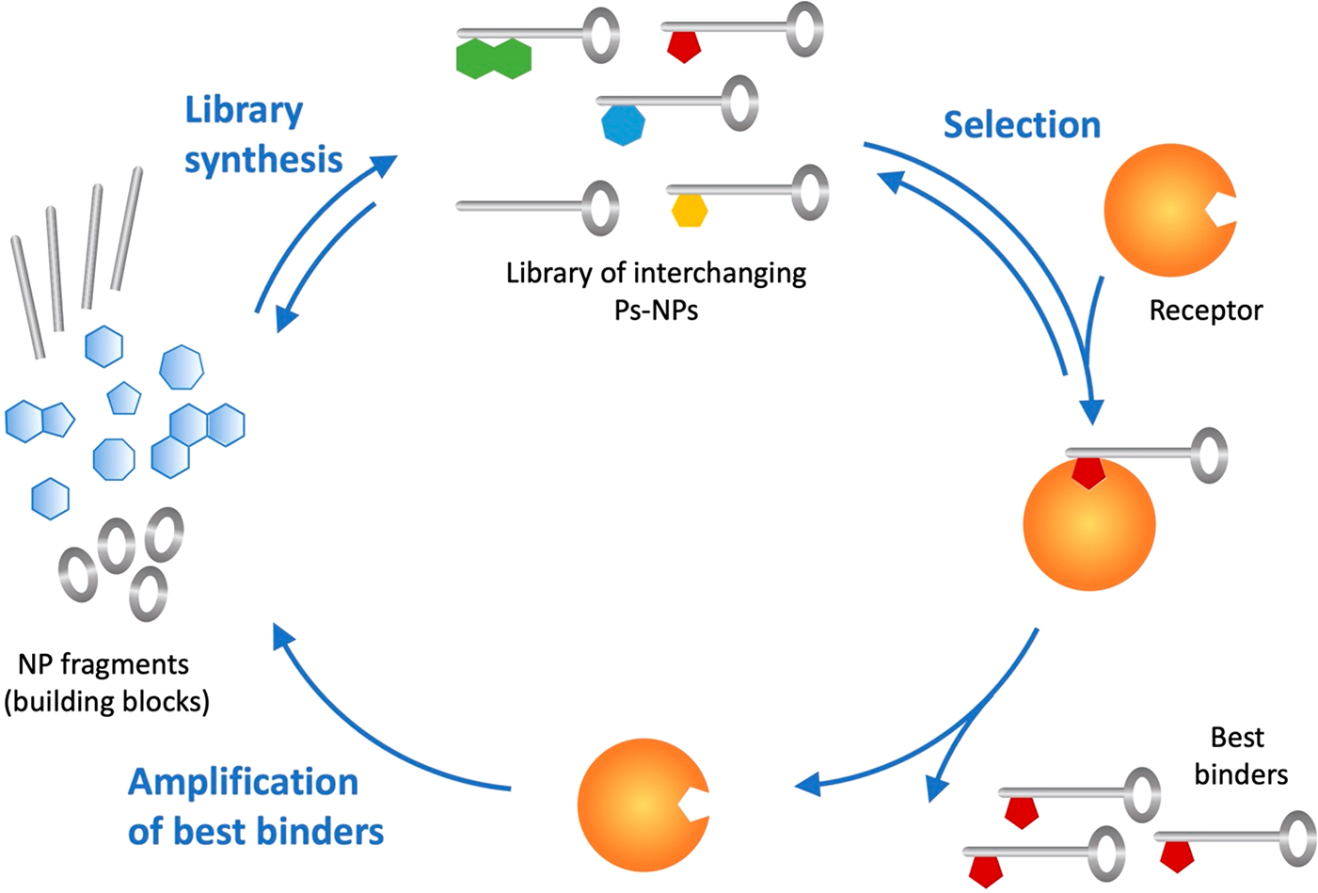
In a dynamic library, potential ligands to a target of interest are synthesized using a set of building blocks (here, NP fragments) and reversible reaction types allowing for continuous interchange. Upon addition of a molecular receptor (usually a protein), the best binders are selected and thereby removed from the equilibrated pool, forcing the dynamic system to respond by resynthesis of this type of small molecule until the equilibrium is reached again.^78^

The immediate usefulness of this concept is demonstrated by the examples described above and those previously reported;^33^ however, it may be possible to improve the iterative optimization strategy by greater involvement of evolutionary aspects by (1) including the target molecule of interest for the *direct selection* of active ligands or inhibitors and (2) allowing for reversible and competitive product formation in compound libraries. Dynamic combinatorial chemistry (DCC) provides for libraries capable of responding to target-binding events by changing their composition, thus offering a chance to improve signals for detection. In the context of pseudo-NP synthesis, DCC will start from pools of soluble, reasonably stable NP fragments (building blocks) that react in a single compartment to form an adaptive library—provided that appropriate functional groups and reversible reaction types are available that give access to continually interchanging adducts. In the presence of a target, such a dynamic process allows for the *amplification* of active molecules within the library, in the sense of a “self-screening process” that was already suggested by J.-M. Lehn (Figure 10).^78^

Although DCC has already become important for biomedical research, it is limited by low library diversity and missing tools for the analysis of large libraries.^79^ A strategy which promises to circumvent these limitations is DNA-encoded library (DEL) synthesis which enables the generation and selection of large-scale dynamic libraries on the basis of DNA-mediated assembly, and facilitates identification of individual compounds by deciphering of a DNA tag which encodes building blocks and reaction steps. Substantial progress in this field^80,81^ may also provide for a basis to dynamic combinatorial libraries of future pseudo-NPs.

## Discussion

### Validation of the Pseudo-Natural Product Concept

The pseudo-NP concept is intended to overcome limitations that are inherent to other NP-inspired molecular design principles and explore new areas of biologically relevant chemical space. The concept merges the biological relevance of NPs with the facile exploration of chemical space of fragment-based discovery by combining NP fragments to obtain scaffolds that retain relevance to NPs but are not possible through current biosynthetic pathways.

The pseudo-NP scaffolds can be made up of the combination of NP fragments that are synthetically made, fragment-sized NPs, or derived from NPs by ring distortion or degradation. Scaffold diversity can be achieved through combining different NP fragments, fusing fragments together with different connectivity patterns, and/or having fragments in different arrangements. We recently investigated whether different pseudo-NP fragment combination principles (as described in Figure 2b) can afford chemically and biologically diverse compound collections. The studies mixed and matched a small set of NP fragments in complementary arrangements^39^ as well as combined the same two NP fragments but with different connectivity patterns and/or fragment orientations.^40^ Evaluation of the pseudo-NPs by cheminformatics and the Cell Painting Assay revealed that the compound sets were indeed chemically and biologically diverse and supports the hypothesis that different fragment combination strategies can lead to diverse compound collections.

**Figure 11:**
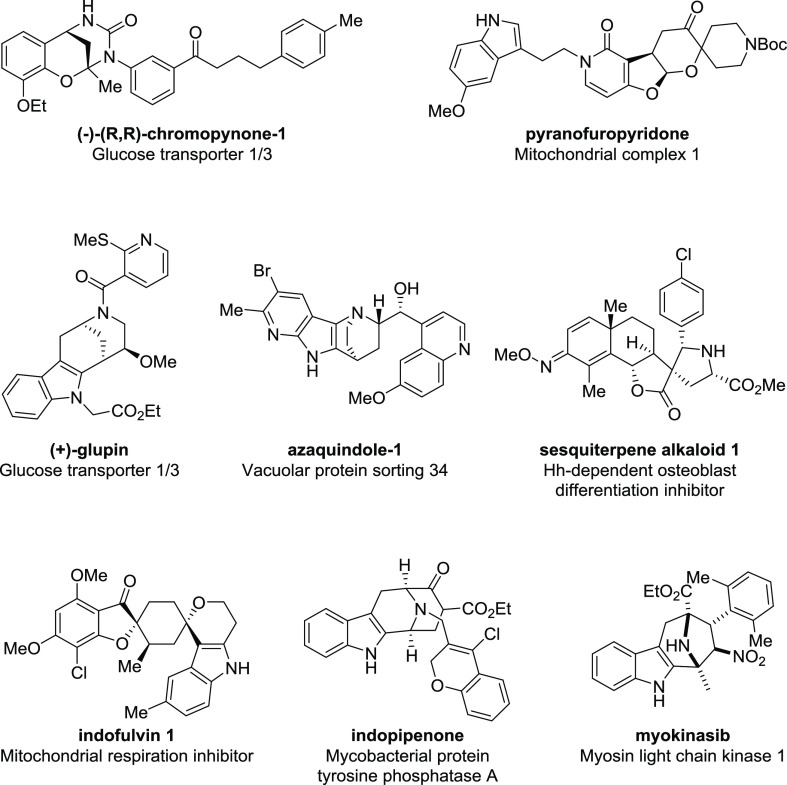
Structures of pseudo-natural products for which biological targets and/or biological modes of action have been determined.

Beyond morphological profiling, we have observed that pseudo-NP collections are enriched with bioactivities that affect therapeutically relevant processes and pathways (Figure 11). Interestingly, many of these examples have concluded that the bioactivity of the pseudo-NP is not due to either individual NP fragment by itself, but rather the combination and the orientation of the NP fragments. These unprecedented NP fragment combinations represent new chemotypes for the identified bioactivities in Figure 11, indicating that design guided by the pseudo-NP concept has led to the exploration of new chemical space while retaining biological relevance. In addition to our own studies, several other research groups have reported the synthesis of bioactive pseudo-NPs.^82−85^

While recent examples reported by us and others delineate the biological potential of pseudo-NP compound design, it should be noted that 21% of all synthetic bioactive molecules (as summarized in ChEMBL) are actually pseudo-NPs.^70^ This shows that vast numbers of pseudo-NPs have been unintentionally synthesized without a guiding principle for decades. The frequent occurrence of bioactive pseudo-NPs supports the concept of pseudo-NPs as a design principle for the generation of novel bioactive small molecules.

From a conceptual perspective, the pseudo-NP workflow represents a chemical evolution of NP structures. Genotypes encoded in biologically relevant NP fragments can undergo “mutations” by chemical alteration and recombination in unprecedented manners that may afford new phenotypes in the form of pseudo-NP scaffolds that can interact with biological systems. “Selection” of pseudo-NPs via biological assays may then lead to further iterations of the cycle to provide more optimal or “evolved” chemical and biological information.

As highlighted here and elsewhere,^33,34^ the pseudo-NP concept has combined fragment-based discovery with the biological relevance of NPs to guide the design of biologically unprecedented compounds. The logic of the concept represents a human-made equivalent to natural evolution and can be viewed as the chemical evolution of NP structure. Taken all together, the pseudo-NP approach represents a validated design principle for the wider exploration of biologically relevant chemical space.

### Design and Prediction of Future Pseudo-Natural Product Classes

Understanding the differences between how chemists and Nature select NP fragments for combination and how they are connected may help guide the design of future compound collections (Figure 6 and Table 1). Although ChEMBL pseudo-NPs contain NP fragments, their molecular features more closely aligned with drug-like compounds than NPs (i.e., low fsp^3^ and nitrogen-rich).^86−89^ Accordingly, it is recommended that future pseudo-NP collections should not focus on combining solely nitrogen-rich and/or aromatic NP fragments, but deviate to incorporate a balance of saturated oxygen-containing and/or aliphatic NP fragments with a maximum total of four NP fragments. Furthermore, NP fragment connectivity patterns should not be so heavily enriched by monopodal connections (77% frequency in ChEMBL pseudo-NPs, Figure 6c). Employing a wider array of fusion patterns that are more frequent in NPs may inherently mimic the broader shape distribution observed in NPs relative to the generally rod- and/or disk-like shapes of synthetic compounds and may also lead to more diverse bioactivity profiles between collections.^90−92^ Access to these desired features will require the use of existing and the development of new complexity-generating reactions in stereoselective and asymmetric fashions rather than the simple linear linkage of fragments. The resulting scaffolds will be enriched in stereochemical content and have high densities of chemical information.^72^

By selecting more sp^3^-enriched and oxygen-containing NP fragments as combination partners and employing a broader distribution of fusion patterns, future pseudo-NP collections may more closely mimic properties Nature has instilled in NPs; i.e., they will be more NP-like.^71^ However, novel arrangements of NP fragments will deviate from the current NP structure to afford compound classes that will explore new areas of chemical space while retaining the biological relevance of NPs.

Beyond having Nature guide pseudo-NP design, the choice of fragment combination may also be influenced by evaluating the biological diversity of current pseudo-NPs. If the arrangement of a common set of fragments results in diverse biological profiles, then the fragments could be classified as nondominating and may be good candidates for future combinations. Conversely, if pseudo-NPs with a common fragment share similar biological profiles, the fragment could be classified as dominating and may lead to redundant bioactivity profiles in future combinations.

We recently analyzed a set of pseudo-NPs comprised of common fragments via the Cell Painting Assay and were able to classify fragments as nondominating or dominating.^39^ These assignments were used to design new pseudo-NP compound classes that were correctly predicted to have unique and similar morphological profiles relative to the initial set of pseudo-NPs. Similar analyses may help guide the design of future NP fragment combinations to obtain biologically diverse compound collections that more efficiently explore biological space.

The initial design of the pseudo-NP concept is to employ NPs as starting points since they are biologically prevalidated compounds selected through natural evolution, as are their fragments.^35^ However, it should be noted that biologically relevant chemical space is not occupied only by various combinations of NP fragments (i.e., NPs, NP derivatives, or pseudo-NPs). This is clear from the large number of bioactive molecules that contain or are exclusively composed of purely synthetic fragments.^93,94^ These man-made fragments in bioactive compounds should therefore also be considered biologically relevant. The evolutionary algorithm of the pseudo-NP concept (Figure 9) may then be used in a broader sense to include not only NP fragments but fragments of all biologically relevant compounds. This may expand the exploration of the concept from evolutionarily relevant NP-like chemical space to more general biologically relevant chemical space.

## Outlook

The pseudo-NP design criteria provide numerous opportunities for molecular discovery programs. Nevertheless, multidisciplinary investigations and advancements are needed to facilitate the exploration of the biologically relevant chemical space that can be accessed by pseudo-NP structure. From a cheminformatic perspective, a further understanding of the differences between how Nature and chemists have selected which NP fragments to combine and how to combine them may aid in the design of future compound collections. At the core of accessing new pseudo-NPs are novel fragment combinations through organic synthesis which will heavily rely on the use and development of complexity-generating methodologies envisioned by organic chemists. Furthermore, the identification of biological relevance of future pseudo-NPs will be underpinned by the continuous development of broad, high content assays and new techniques for target identification by biologists and chemical biologists. Through multidisciplinary collaboration and advancements, the design, synthesis, and biological evaluation of new pseudo-NPs may help navigate biologically relevant chemical space to provide compounds that will be beneficial to chemical biology and medicinal chemistry programs.

## Supporting Information Available

Computational details of the data provided in Table 1 and definitions of genotype, phenotype, evolution, and information in different contexts (PDF)
