# Supplementary material for: Chemical Evolution of Natural Product Structure
Source: J Am Chem Soc. 2022 Feb 21;144(8):3314–29. doi: 10.1021/jacs.1c11270 (PMC8895405; doi:10.1021/jacs.1c11270)
Supplement: Supplementary file 1 — ja1c11270_si_001.pdf [file ja1c11270_si_001.pdf]

Supporting Information

Chemical Evolution of Natural Product Structure

Michael Grigalunas[a], Susanne Brakmann[b], and Herbert Waldmann[ab]*

[a]Max-Planck-Institute of Molecular Physiology, Otto-Hahn Strasse 11, 44227, Dortmund, Germany

[b]Faculty of Chemistry and Chemical Biology, TU Dortmund University, Otto-Hahn Strasse 4a, 44227, Dortmund, Germany

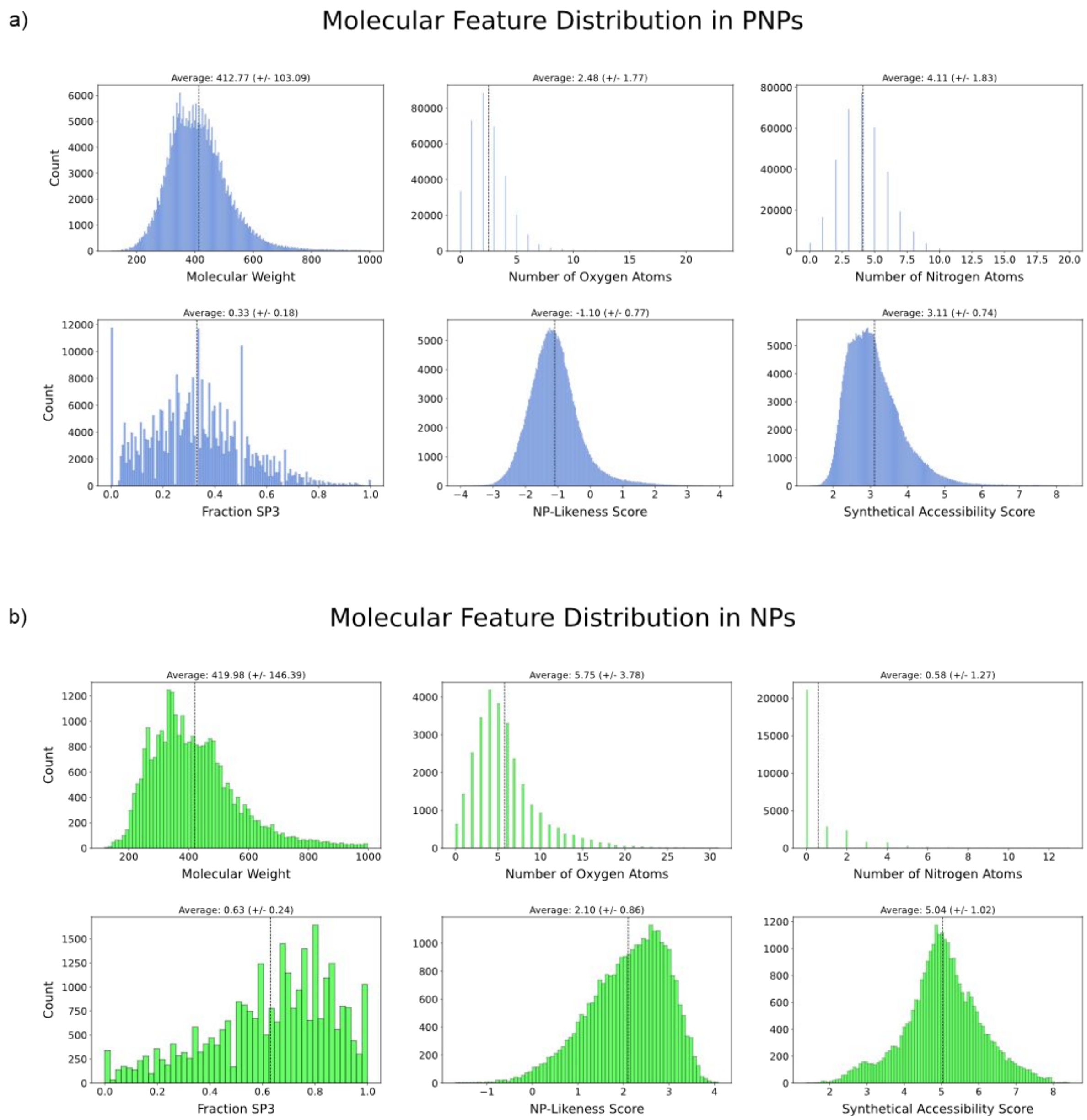

SI Figure 1: Molecular feature distributions in a) ChEMBL pseudo-NPs and in b) NPs. The data sets used were obtained from Waldmann et al.[1]

Definitions

Genotype

Classical: The totality of an organism's hereditary makeup; at the molecular level, synonymous with the totality of genetic information encoded in the form of nucleotide sequences of DNA).

Molecular theory of natural evolution: *Genetic information* is contained in nucleic acids (DNA or RNA) and encoded with the nucleobases A, G, C, and T (U) which serve as molecular symbols. It results from a process that assesses "replication frequency", "replication quality" and "life span" of a sequence of symbols by favoring sequences with the best performance values – the "fittest", in Darwianian sense. A characteristic molecular feature of genetic information is that its reading and decoding requires molecular recognition based on complementarity of the molecular symbols.

Chemical evolution of PNP: Molecular structures encode *chemical information* using symbols that represent the microenvironment of every atom with valence electrons, number of bonds, number of bound non-H atoms, and various symmetry properties. The information inherent to a molecule or, its molecular complexity, results additively from these symbols.[2,3] Regarding PNPs, a further level adds to the general chemical information content: They encode biologically relevant information with symbols, NP fragments, that have been *selected* during natural evolution based on their contribution to increasing an organism's "fitness". This is a true genetic information that is inheritable because it confers specific, selected molecular recognition properties to descendants.

Phenotype

Classical: The totality of characteristics of a fully developed individual or, its observable "appearance". It results from interactions of genetic information with internal and external influences during ontogeny (i.e., the development of an individual organism from embryo to adult). In unicellular organisms, the term "phenotype" is also used to refer to characteristics of separate individuals of a strain.

Molecular theory of natural evolution: Feedback loops exist that link the nature and behavior of an organism (its "fitness"; see "Genotype, classical definition") to the sequence of symbols it harbors. The coding sequence has a "meaning" that is *expressed* (= becomes evident) in the form proteins which contain a direct (= colinear) *translation* of the nucleic acid sequence, however, having the ability to fold into three-dimensional structures exhibiting a greater spectrum of specific chemical properties

and functions such as the ability to catalyze, to recognize and bind, to stabilize or move other molecules, and others.[3]

Chemical evolution of PNP: NPs, NP fragments and PNPs form three-dimensional structures whose topology, symmetry or asymmetry, polarity, acidity, etc. are a direct consequence of their composition of symbols. The totality of their chemical properties expresses their appearance as evident by their repertoire to recognize and react.

Evolution

Classical: "Biological evolution" refers to changes in the set of characteristics of living organisms in the succession of generations. It takes place in populations and is controlled by selection processes on *phenotypes* (see above): the latter evaluate the organism's adaptation to its environment/to selective constraints.

Molecular theory of natural evolution: In the most general view, the essential conditions for evolutionary behavior require reproduction (or self-reproduction) and mutation. This may be observed with molecules. Regarding Darwinian evolution, metabolism, self-reproduction and mutation are necessary elements of the (iterative) process.[4]

Chemical evolution of PNP: The general view of a "chemical evolution" assumes that the gradual formation of biomolecular building blocks on primordial Earth before the onset of "biological evolution" started from components of the primordial atmosphere (methane, hydrogen, ammonia), first products derived from these (formaldehyde, hydrogen cyanide, etc.) and the primordial oceans together with available forms of energy. The idea of a chemical evolution of PNP extends this concept by allowing for increasing molecular complexity by "mutation" during combinatorial synthesis (*in vitro*).

Information

Classical: A universal definition refers to information as a "symbolically encoded, abstractly represented message that conveys an expected action and an intended purpose".[5] Information is exchanged between an (intelligent) sender and an (intelligent) receiver on five levels of "communication":

- Statistics: transmitted signal vs. received signal
- Code and syntax: code that was used vs. code that was understood
- Meaning: assigned meaning vs. determined meaning
- Action: expected action vs. executed action
- Purpose: intended purpose vs. achieved purpose

Shannon's theory describes the information content of a message using entropy H:

$$H = -\sum_i p_i log_x p_i$$

Here, p_i is the probability for an event *i*, and *x* is arbitrary – typically 2. The unit is *bit*.

Chemical information: "The intrinsic complexity of a molecule can be calculated, based on the principles of information theory, from the information content of the chemical microenvironment of each atom of a molecule".[2] – Discrete variables are defined that reflect the number of degrees of freedom, including the number of valence electrons of an element at position *i* (= V_i), number of bonds b_i, number of different non-hydrogen elements at position *i* and its direct neighbors (e_i), number of different (non-hydrogen) substituents at a stereogenic atom (d_i), and number of isomeric arrangements (s_i). These numbers sum up to *molecular comple*xity

$$C_m = \sum_i d_i e_i s_i log_2(V_i b_i)$$

having the unit *mcbit* ("Böttcher scores").[1] Using this definition, chemical synthesis can be considered as process for encoding information.[2] With comparative compilation of molecular complexities according to Böttcher, Shenvi showed that secondary metabolites encode information differently from other complex structures such as dendrimers, dyes, and drugs, because they contain a larger number of sp^3-hybridized atoms, a high number of stereogenic atoms, a high number of heteroatoms, and low aromaticity.[2] This can be attributed to a high *information density*, measured in *mcbits/Å³*, which is particularly high for natural products. For numerical values of $C_m/Å^3$, see Shenvi, Fig.2,[2] for C_m/M, see Böttcher, Fig. 5.[1]

Information encoded by NPs and PNPs: Beyond molecular complexity and information density, *biological relevance* adds to the information content of these molecules. Secondary metabolites are synthesized because they are biologically relevant and confer a reproductive advantage to the organism. According to C. F. von Weizsäckers's statement that "information is what is understood", biological relevance can be translated to "molecules which transport a message that can be understood" – in molecular terms, which can bind and be recognized.

Computational details for the cheminformatic analysis of ChEMBL pseudo-NPs and NPs

The following data was produced according to Gally *et al.*[1] with the NPFC package (v. 0.7.8). Structural information from ChEMBL (ChEMBL 26, 1,941,411 records) and DNP (DNP291_ct_no8, 318,271 records) was extracted from SDF files. Compounds were converted to RDKit format (v. 2020.09.1) and then standardized by applying the following steps: filter empty structures, disconnect metal atoms, keep only the largest organic entity in mixtures, apply deglycosylation, clear isotopes, normalize functional groups, remove formal charges, enumerate canonical tautomer and clear stereochemistry information. Additionally, a set of filters was applied after deglycosylation to remove compounds with unwanted features based on different criteria: number of heavy atoms (x < 4), molecular weight (x > 1000.0 Da), number of rings (x < 1), chemical elements (not H, B, C, N, O, F, P, S, Cl, Br or I). Then, duplicates from each dataset were removed separately via identity of InChI Keys and 2D coordinates were computed for the resulting unique molecules. Details of each of the steps can be found in the official documentation of the NPFC package: https://npfc.readthedocs.io/en/latest/ex_preparation.html. Finally, NPs were removed from the ChEMBL data set using the InChI Keys from DNP as reference.

A data set of 2000 fragments, originally produced by Over *et al.*[6] was downloaded as SDF and prepared using a similar protocol (but without filter and deglycosylation) and from which Murcko scaffolds were extracted. The benzene fragment was removed from the results due to being ubiquitously found in both synthetic and natural datasets. The resulting 1673 structures were then used as substructure search for querying the prepared DNP (165,467) and ChEMBL (1,632,769) compounds. Fragment combination analysis was performed on the molecules with at least 2 NP-derived fragments. Fragment combination graphs were subsequently generated using the fragment combination information. Finally, the DNP graphs (representing 28,386 compounds) were used as a representation of the NP-derived fragment connectivity to identify the 344,394 PNPs in the 437,071 remaining structures in ChEMBL.

The molecular properties and NP-likeness scores[7] of remaining molecules in DNP and the PNPs in ChEMBL were then computed using RDKit.

References

(1) Gally, J.-M.; Pahl, A.; Czodrowski, P.; Waldmann, H. Pseudo-Natural Products Occur Frequently in Biologically Relevant Compounds. *J. Chem. Inf. Model.* **2021**, *61*, 5458.

(2) Böttcher, T. An Additive Definition of Molecular Complexity. *J. Chem. Inf. Model.* **2016**,

(3) Demoret, R. M.; Baker, M. A.; Ohtawa, M.; Chen, S.; Lam, C. C.; Khom, S.; Roberto, M.; Forli, S.; Houk, K. N.; Shenvi, R. A. Synthetic, Mechanistic, and Biological Interrogation of Ginkgo Biloba Chemical Space En Route to (-)-Bilobalide. *J. Am. Chem. Soc.* **2020**, *142*, 18599.

(4) Eigen, M.; Winkler-Oswatitsch, R. *Steps Towards Life: A Perspective on Evolution*; Oxford University Press, 1996.

(5) Gitt, W.; Compton, R.; Fernandez, J. *Biological Information — What Is It?*; Marks II, R. J., Behei, M. J., Dembski, W. A., Gordon, B. L., Sanford, J. C., Eds.; World Scientific: Singapore, 2013.

(6) Over, B.; Wetzel, S.; Grütter, C.; Nakai, Y.; Renner, S.; Rauh, D.; Waldmann, H. Natural-Product-Derived Fragments for Fragment-Based Ligand Discovery. *Nat. Chem.* **2013**, *5*, 21.

(7) Ertl, P.; Roggo, S.; Schuffenhauer, A. Natural Product-Likeness Score and Its Application for Prioritization of Compound Libraries. *J. Chem. Inf. Model.* **2008**, *48*, 68.
